# An Eco-Friendly Disposable Plasmonic Sensor Based on Bacterial Cellulose and Gold †

**DOI:** 10.3390/s19224894

**Published:** 2019-11-09

**Authors:** Nunzio Cennamo, Carlo Trigona, Salvatore Graziani, Luigi Zeni, Francesco Arcadio, Giovanna Di Pasquale, Antonino Pollicino

**Affiliations:** 1 Department of Engineering, University of Campania Luigi Vanvitelli, Via Roma 29, 81031 Aversa, Italy; nunzio.cennamo@unicampania.it (N.C.); luigi.zeni@unicampania.it (L.Z.); francesco.arcadio@unicampania.it (F.A.); 2 Department of Electrical, Electronics and Computer Engineering (DIEEI), University of Catania, Viale Andrea Doria 6, 95125 Catania, Italy; salvatore.graziani@dieei.unict.it; 3 Dipartimento di Scienze Chimiche, University of Catania, Viale Andrea Doria 6, 95125 Catania, Italy; giovanna.dipasquale@dii.unict.it; 4 Department of Civil Engineering and Architecture, University of Catania, Viale Andrea Doria 6, 95125 Catania, Italy; apollicino@unict.it

**Keywords:** optical sensors, sustainable development, localized surface plasmon resonance, bacterial cellulose, eco-friendly disposable sensors

## Abstract

In several application fields, plasmonic sensor platforms combined with bio-receptors are intensively used to obtain biosensors. Most of these commercial devices are based on a disposable chip. Usually a gold chip, functionalized with a specific bio-receptor, inside a costly sensor system, is used. In this work, we propose a low-cost and small-size sensor system, used for monitoring a disposable plasmonic chip, based on an innovative optical waveguide made of bacterial cellulose (BC). In particular, we have sputtered gold on the green slab waveguide that is able to excite localized surface plasmon resonance (LSPR). Experimental results are presented on the capabilities of using the BC-based composite as an eco-friendly plasmonic sensor platform, which could be exploited for realizing disposable biosensors. The sensor has been used with optical fibers and simple equipment. More specifically, the fibers connect the green disposable LSPR sensor with a light source and with a spectrometer. The novel plasmonic sensing approach has been tested using two different optical waveguide configurations of BC, with and without ions inside BC.

## 1. Introduction

Innovative biosensors in optical fibers are capable of on-site and real-time monitoring of different substances, by exploiting phenomena such as surface plasmon resonance (SPR) or localized surface plasmon resonance (LSPR). The plasmonic optical fiber sensors can be used for remote sensing and can reduce the dimensions and price of plasmonic sensor systems. As such, they are used, combined with several kinds of receptors, in many research fields [1,2,3,4,5]. 

Many configurations of plasmonic optical fiber sensors have been proposed in order to improve the performances in terms of throughputs, reliability, robustness, and miniaturization [6,7,8,9,10,11,12,13,14]. Usually, the optical fiber sensors are defined as intrinsic and extrinsic. In particular, when the interaction of the fiber with the analyzed medium is present, they are defined as intrinsic optical fiber sensors, whereas, when the optical fiber is used only as a mere waveguide allowing the launching of the light to the sensing area and its collection, they are defined as extrinsic optical fiber sensors. The advantage of extrinsic optical fiber sensors are the capability of the remote sensing, typical of the optical fibers, and the possibility of exploiting the input and output fibers only to connect the equipment with the sensor chip, usually disposable, low-cost, and with a better reproducibility from chip to chip [12,13].

In this work, an extrinsic green plasmonic optical fiber sensor, based on an eco-friendly disposable plasmonic chip made of bacterial cellulose (BC), is proposed. Preliminary results on this innovative and green approach of sensing have been previously presented [15]. Here, we have realized and tested two extrinsic plasmonic optical fiber sensors, based on two different slab waveguides of BC, with and without ions inside, covered by gold, examining in depth all the aspects. Finally, we have also compared these two innovative green extrinsic plasmonic optical fiber sensors. 

Nahid Pourreza et al. [16] have presented an innovative plasmonic sensor, exploiting a BC thin film, with embedded silver nanoparticles, but their approach (a BC nano-paper, with silver nanoparticles) differs from the one proposed in this work, mainly because the BC layer is a micro-paper and the metal (gold in our case) covers the cellulose wires, configuring a kind of nanowires able to excite LSPR.

Cellulose is the most ambulant organic polymer present in nature. It is biocompatible and fully biodegradable [17]. Eventually, it can be processed to be used in “non-conventional” flexible and green electronics. Cellulose is, generally, obtained by plant sources by the pulp industry. Unfortunately, the extraction of cellulose requires relevant amounts of energy and freshwater. Eventually, waste products are obtained, raising pollution issues [18]. This production process, the appeal of cellulose as a green raw material, is greatly reduced. BC is a kind of cellulose obtained by totally different processes. BC is, actually, produced at the interface between the open air and a suitable culturing medium [19]. Growing BC is possible in typical laboratory environmental conditions. No relevant quantities of energy and water are required. Because of the described production procedure, BC is considered as a greener alternative to plant-derived cellulose. Not less importantly, BC is produced in a much purer form than cellulose derived from plant sources. 

As a consequence of the considerations reported above, a growing interest has been devoted to BC as a base component for realizing green transducers. Recently, some of the authors have shown the possibility of using BC for realizing mechano-electric transducers, exploiting the presence of ionic liquids (ILs) inside the compound [20,21]. Here, we have tested the effect of the presence of ILs in BC. More specifically, the influence of the thickness and the dimensions of the BC papers (the optical waveguide) on the optical responses in plasmonic sensing has been investigated.

## 2. Materials and Methods

### 2.1. The Bacterial Cellulose Papers With and Without ILs

The bacterial cellulose used in this study was provided by BioFaber, and produced by bacteria that feed on food waste. In this way, an eco-sustainable and nanostructured material has been obtained, having the same chemical structure as the vegetable-derived cellulose, that is, consisting of long chains in which the glucose units are bound together by β (1–4) glycosidic bonds.

Through thermogravimetric analysis it was determined that the samples of BC, in equilibrium with the air, contain a percentage of water equal to 4%.

**Figure 1:**
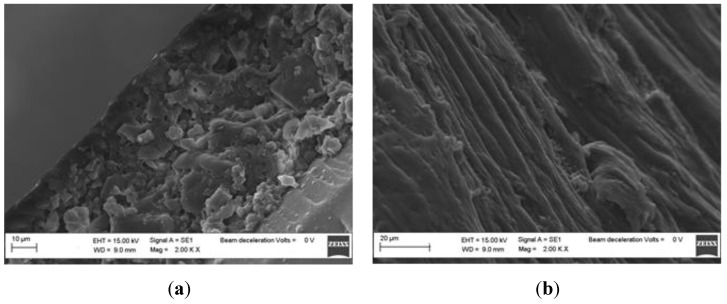
Section of a bacterial cellulose sample (**a**); Section of a sample of bacterial cellulose after treatment with ILs (**b**).

The IL adsorbed on the cellulose was 1-Ethyl-3-Methylimidazolium tetrafluoro borate (EMIM-BF4), purchased from Alfa Aesar and used without further purification. Absorption was achieved by immersing a sample of bacterial cellulose in the IL, inside a dryer containing anhydrous calcium chloride, for 24 h. At the end of this period, the sample (BC-EMIM-BF4) was kept under vacuum at 60 °C for a further 24 h. Through gravimetric measurements it was therefore determined that the amount of adsorbed IL is equal to 34% by weight. SEM micrographs have shown that the treatment of BC with the IL determines a variation of the morphology due to the partial solubilization of the cellulosic nanostructures (Figure 1a,b).

### 2.2. The Plasmonic Sensors Based on BC With and Without ILs

Gold was sputtered on the top of both BC slab waveguides in order to realize the plasmonic sensors, with and without ILs. We have used two samples of BC (one impregnated with ILs and one without any IL inside) with the same thickness, about 0.14 mm, and the same dimension, about 1 cm × 2 cm. In particular, the gold has been sputtered on the top of both the BC papers by using a sputtering machine (Bal-Tec SCD 500, Bal-tec AG, Balzers, Liechtenstein). The sputtering process has been repeated three times, with a current of 60 mA for 35 s (depositing 20 nm per step), covering the cellulose wires and configuring a kind of nanowires able to excite LSPR. In fact, as shown in Figure 1, the mesh of the BC layers is in a micrometer scale. When the sputtering process is used for the gold nano-deposition, the cellulose nanowires are covered by gold, without obtaining a continuous gold film.

**Figure 2:**
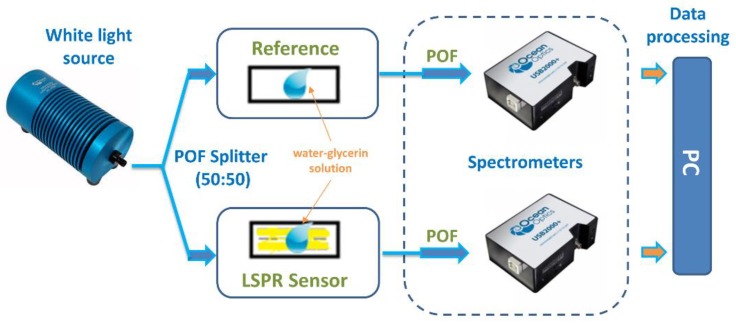
Outline of the experimental setup used to test the plasmonic sensors.

The eco-friendly disposable LSPR sensor platforms have been characterized using a very low-cost, small size, and simple experimental setup, shown in Figure 2 and based on:

- a halogen lamp (HL–2000–LL, Ocean Optics, Dunedin, FL, USA), used as white light source;
- a plastic optical fiber (POF) coupler (50:50) connected with this source;
- two green slab waveguides of BC with the same dimensions, one covered by gold (LSPR sensor) and one without gold (reference);
- two POFs connecting the slab waveguides of BC with two similar spectrometers (USB2000+UV–VIS spectrometer, Ocean Optics, Dunedin, FL, USA).

More specifically, from the datasheet of Ocean Optics, the spectral emission of the light source ranges from 360 nm to 1700 nm and both the spectrometers are sensitive from 300 nm to 1050 nm with a spectral resolution of the spectrometer of 1.5 nm (full width at half maximum (FWHM)) [11,12,14]. 

For different water-glycerin solutions contacting the sensors, the LSPR spectra were obtained by the transmitted spectra of the sensor normalized on the transmitted spectra of the reference channel (obtained by the slab waveguide (BC paper without gold) with the same surrounding medium).

## 3. Experimental Results

To test and to compare the LSPR platforms based on BC papers, with and without ILs, different water–glycerin solutions in contact with the LSPR slab waveguides were used to change the refractive index and obtain the bulk sensitivity of the sensors. These water–glycerin solutions have been tested by an Abbe refractometer (model RMI by Exacta Optech, Germany).

**Figure 3:**
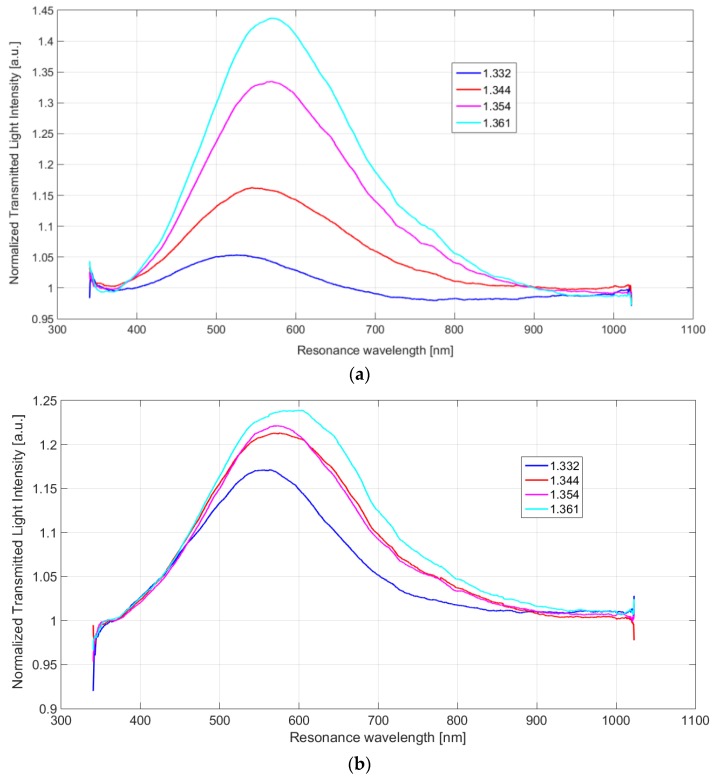
LSPR spectra obtained by two green plasmonic sensor configurations: (**a**) Sensor configuration without ILs in the BC layer. (**b**) Sensor configuration with ILs in the BC layer.

For both the LSPR sensor configurations based on a BC paper with and without ILs, the experimentally obtained LSPR normalized transmitted spectra are shown in Figure 3, for several water–glycerin solutions, with refractive index ranging from 1.332 to 1.361. 

As well as for others LSPR sensors, in both configurations (with and without ILs) when the refractive index changes, the intensity value and the resonance wavelength value in the LSPR spectrum changes also. In particular, as clearly shown in Figure 3 for both cases, when the refractive index increases, the LSPR wavelength shifts on the right (increases), whereas the intensity increases.

The sensitivity (*S*) and the resolution (Δ*n*) of the sensor are two parameters used for the analysis of the performances. In this work, we can define these parameters as a function of the measured value (*M*), the intensity or the resonance wavelength, and of the refractive index of the sensing layer (*n*). So, when the refractive index of the sensing layer “*n*” is altered by “*δn*”, the measured value “M” changes by “*δM*”. It follows that the sensitivity and the resolution of the LSPR sensor can be defined as:

(1) $$S=\frac{\delta M}{\delta n}$$

(2) $$\Delta n=\frac{\delta n}{\delta M}\;\delta M_{meas}=\frac{1}{S}\;\delta M_{meas}$$

where “*δM_meas_*” is the spectral resolution of the spectrometer when we use the resonance wavelength as measured value, and the max experimentally measured variation of the intensity at the resonance wavelength in the other case. 

In particular, in this work we have used for the measured value (*M*) the resonance wavelength value (*ʎ*) or the normalized transmitted light intensity value at the resonance wavelength (*I*). 

From the datasheet of the spectrometer, “*δM_meas_*” is equal to 1.5 [nm] (*δʎmeas*) when the resonance wavelength value is used as measured value (it is the resolution of the spectrometer at FWHM) [11,12,14], whereas it is equal to 0.004 [a.u.] for the sensor based on BC with ILs and it is equal to 0.001 [a.u.] for the sensor based on BC without ILs (in these cases it is the max experimentally measured variation of the intensity at the resonance wavelength (*δImeas*)).

**Figure 4:**
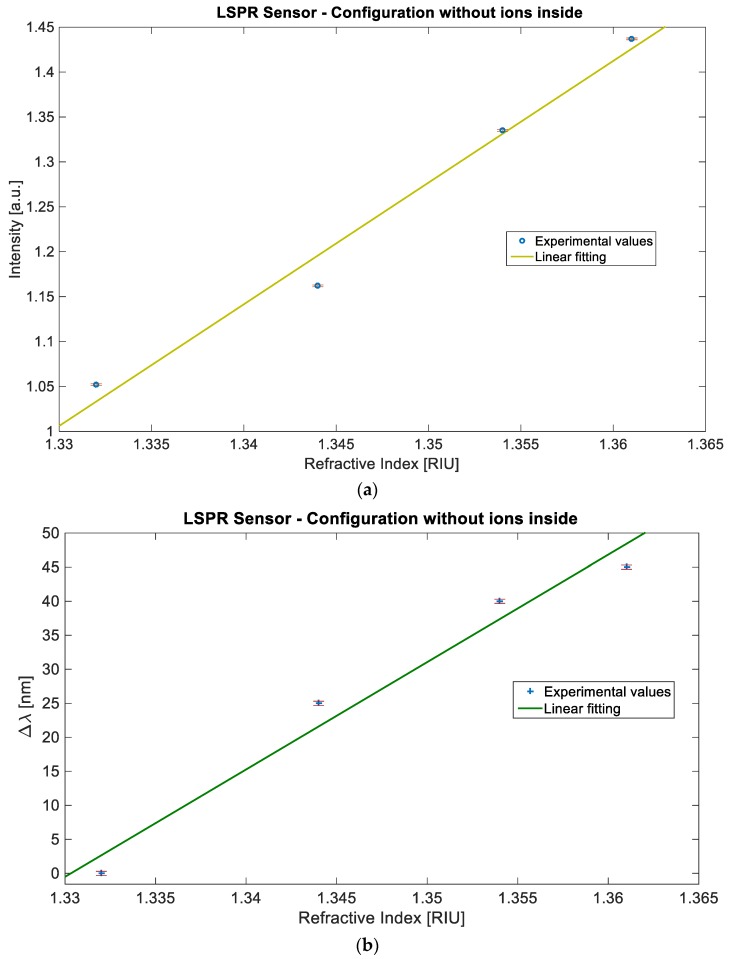
Experimental results of the sensor based on BC paper without ions: Measured values versus the refractive index, with the error bars and the linear fitting of the data. (**a**) Intensity value versus refractive index. (**b**) Resonance wavelength shift value versus refractive index.

For the sensor based on BC paper without ILs, Figure 4a shows the normalized transmitted light intensity value at the resonance wavelength (*I*) versus the refractive index, whereas Figure 4b shows the resonance wavelength variation (Δ*ʎ*) versus the refractive index. In Figure 4a,b the error bars are also presented, and the linear fitting to the experimental data for both measured values. The Pearson’s correlation coefficient (R) is equal to 0.991 for the intensity and 0.985 for the resonance wavelength, showing a good linearity for the sensor’s responses. It is important to underline that the linear fitting is a way to extrapolate a trend and allow an easy comparison between the sensors.

**Figure 5:**
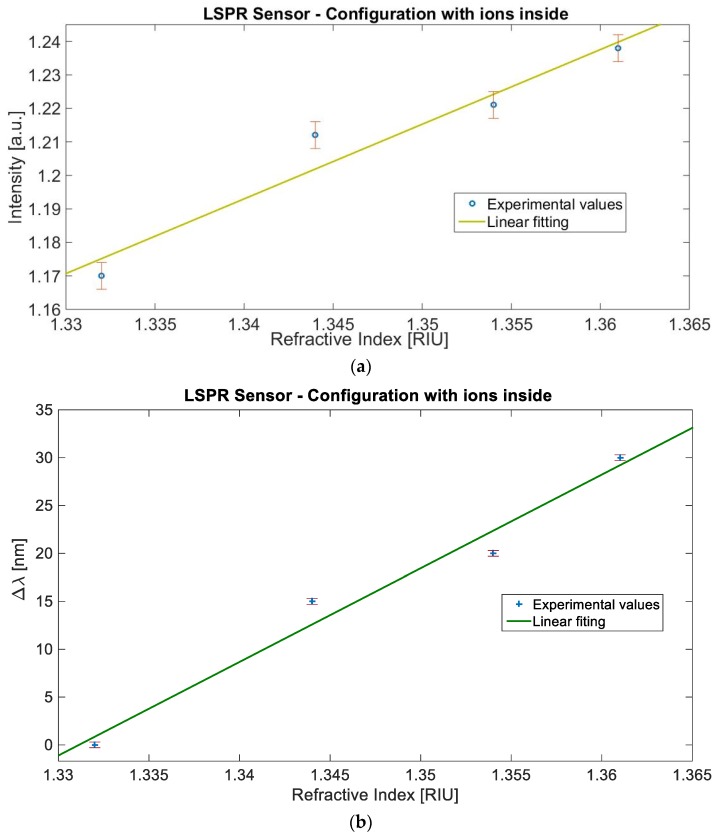
Experimental results of the sensor based on BC paper with ions: Measured values versus the refractive index, with the error bars and the linear fitting of the data. (**a**) Intensity value versus refractive index. (**b**) Resonance wavelength shift value versus refractive index.

In a similar way, for the LSPR sensor configuration relative to BC paper with ILs inside, Figure 5a shows the normalized transmitted light intensity value at the resonance wavelength (*I*) versus the refractive index with the error bars and the linear fitting of the data, similarly, Figure 5b shows the resonance wavelength variation (Δ*ʎ*) versus the refractive index, the error bars, and the linear fitting to the experimental data. Also, in this configuration, the sensor shows a good linearity, as demonstrated by the Pearson’s correlation coefficient (R). In fact, it is equal to 0.971 for the intensity and 0.986 for the resonance wavelength.

## 4. Discussion

From the Equations (1) and (2), the resolution of the sensor (Δ*n*) is the minimum amount of change in refractive index detectable by the sensor, whereas the sensitivity (*S*) is the variation in resonance wavelength or intensity per unit change in refractive index.

More specifically, from the Equation (1), considering that the sensitivity is the change of the measured value per unit change in refractive index, an approximate value of the sensitivity is the angular coefficient of the linear fitting reported in Figure 4 and Figure 5, for both sensor configurations and for both the measured values (*I* or *ʎ*). 

For a clearer comparative analysis between these two sensor configurations, with and without ILs in the BC, Table 1 summarizes the average values of the experimentally measured performance parameters, for external medium refractive index ranging from 1.332 to 1.361.

**Figure 6:**
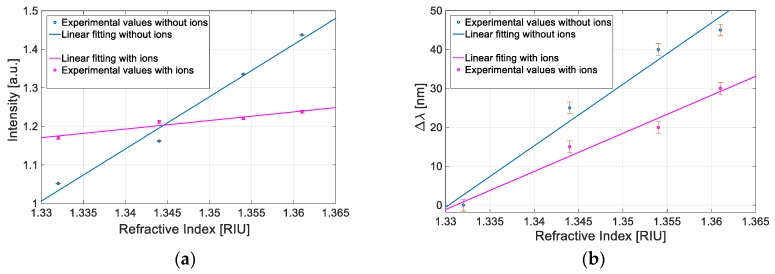
Comparative analysis between two configurations based with and without ILs: (**a**) Intensity value versus refractive index. (**b**) Resonance wavelength shift value versus refractive index.

To compare these results, we have also reported in Figure 6 the measured values (*I* or *ʎ*) versus the refractive index obtained by both configurations with and without ILs.

We have considered a very large refractive index range (from 1.332 to 1.361), so the linear fitting does not imply an actual linear relationship but it has been used to compare the performances; consequently, the calculation of the single values of above-mentioned parameters has been carried out by employing a first-order approach.

As reported in Table 1 and as shown in Figure 6, the experimental results show that the configuration without ILs in the BC paper exhibits better performances in terms of sensitivity and resolution for both the measured values (*I* or *ʎ*).

**Figure 7:**
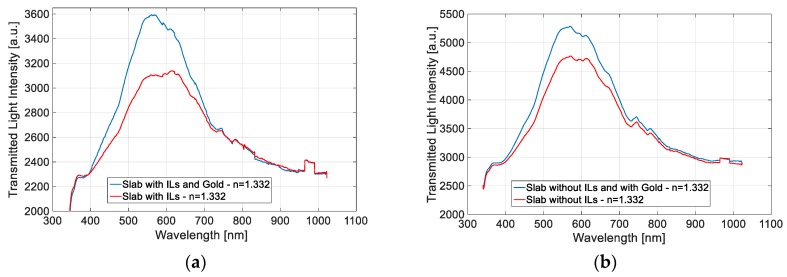
Transmitted spectra in water (*n* = 1.332) obtained before the normalization: (**a**) Slab with ILs. (**b**) Slab without ILs.

We suppose that the configuration with ILs in the BC presents the worst performances because the ILs reduce the light intensity in the slab waveguides (the optical losses increase due to backscattering) and so the interaction with the LSPR is less effective. For this purpose, in Figure 7 we show the transmitted spectra obtained in water (*n* = 1.332) of both configurations (with and without ILs) before the normalization.

In the future, we could reduce the thickness of the BC paper to exploit light-scattering, induced by the ILs in the BC waveguide, to improve the plasmonic phenomenon. In fact, in a waveguide of reduced thickness, the scattered light can increase the amount of energy coupled to the plasmons instead of simply representing a net loss in the transmitted intensity. This can lead to an improvement of the performances in terms of sensitivity and resolution [11].

The advantages of this approach, based on an extrinsic POF sensor, is the possibility of remote sensing by two POFs combined with a removable, eco-friendly, and disposable chip sensor for biochemical sensing applications. In fact, as shown in Table 1, the bulk sensitivity and the resolution of the sensor configuration without ILs in the BC paper are very similar to those obtained by other refractive index sensors (several already tested with receptors for the selective detection of analytes) [1,2,3,4,5,6,7,8,9,10,11,12,13,14,22,23,24,25,26,27,28,29], but with all the advantages of an eco-friendly disposable chip.

## 5. Conclusions

Two novel green, low-cost, easy-to-realize, plasmonic sensors, based on BC papers covered by gold, have been realized and experimentally tested: the first one without ions in the BC paper and the second with ions inside. The experimental results have demonstrated good performances in terms of sensitivity and resolution for both the configurations, when the normalized transmitted light intensity value has been considered, with the best performances in the configuration without ions in the BC paper. The sensor’s performances (Δ*n* ~ 10^−4^) obtained in the best case have demonstrated that this approach can be used for biochemical sensing applications when a self-assembled monolayer bio-receptor is used, in the same way as [24]. 

## Figures and Tables

**Table 1:** Performances comparison for the sensor configurations (with and without ions in BC paper).

Sensor Configuration	S_I_ [a.u./RIU]	Δn _I_ [RIU] = (1/S_I_) × δImeas	S_λ_ [nm/RIU]	Δn _λ_ [RIU] = (1/S_ʎ_) × δʎmeas
BC paper without ions	13.54	7.4 × 10^−5^	1600	9.4 × 10^−4^
BC paper with ions	2.23	1.79 × 10^−3^	980	1.5 × 10^−3^
